# Prognostic value of temporalis muscle thickness as a marker of sarcopenia in intracerebral hemorrhage

**DOI:** 10.3389/fneur.2025.1564550

**Published:** 2025-05-16

**Authors:** Waldemar Gubarev, Jan Klinke, Ulrike Voßmann, Daniel Cantré, Bijan Zendeh Zartoshti, Artem Rafaelian, Milos Arsenovic, Daniel Dubinski, Sae-Yeon Won, Florian Gessler, Thomas Freiman, Alexander Storch, Matthias Wittstock

**Affiliations:** ^1^Department of Neurology, University Medical Center Rostock, Rostock, Germany; ^2^Institute of Diagnostic and Interventional Radiology, Pediatric Radiology and Neuroradiology, University Medical Center Rostock, Rostock, Germany; ^3^Department of Neurosurgery, University Medical Center, Rostock, Germany

**Keywords:** intracerebral hemorrhage, prognosis, sarcopenia, temporal muscle thickness, modified ranking scale

## Abstract

**Introduction:**

Estimating the prognosis of spontaneous intracerebral hemorrhage (ICH) is of great importance. It has not been conclusively clarified whether sarcopenia is predictive for the functional outcome in ICH. Determining the temporalis muscle thickness (TMT) may be helpful for estimating sarcopenia. An association of TMT with outcome (mRS) has been shown in cerebellar ischemia and traumatic brain injury.

**Methods:**

The present retrospective study of 488 consecutive patients with ICH aimed to investigate the association of sarcopenia as assessed by TMT with mRS. In addition to biometric data, ICH subtype and severity [modified ICH score (mICH)], occurrence of complications and mRS at discharge and after 90 days were recorded. The influence of sarcopenia assessed by TMT as the surrogate marker using head imaging (cCT, cMRT) on mRS was analyzed by ordinal regression analysis. Dichotomization into sarcopenic and non-sarcopenic patients was carried out using standard threshold values.

**Results:**

Finally, 322 patients were analyzed [median (IQR) age: 77 (66–83) years; 57.5% male]. Sarcopenic patients were older (*P* < 0.001), had lower BMI (*P* = 0.025) and higher mICH scores (*P* < 0.001) compared to non-sarcopenic patients. There was no significant difference in the overall distribution of mRS scores between sarcopenic and non-sarcopenic patients at discharge (unadjusted common OR: 1.28; 95% CI: 0.85–1.92; *P* = 0.236), but at 90 days favoring the non-sarcopenic over the sarcopenic group (unadjusted common OR: 1.41; 95% CI: 1.07–2.12; *P* = 0.049). The results did not subsist statistical adjustment to candidate covariates by multivariate ordinal regression.

**Discussion:**

In conclusion, sarcopenia as assessed by TMT seems to have limited prognostic value in ICH.

## Introduction

Spontaneous intracerebral hemorrhage (ICH) accounts for 10–15% of all stroke with mortality up to 61% at 1 month after bleeding (1, 2). Given severe neurological dysfunction and impaired consciousness, ICH survivors often require admission to the intensive care unit (ICU) or neurological ICU (NICU) (3). Along with this, incidence of ICH increases with higher age (4). Despite improvements in care and treatment, functional outcome in those elder patients with ICH is not always as favorable as expected. Therefore, additional efforts to identify novel prognostic markers and improve patient outcomes are required. Sarcopenia, defined as the loss of skeletal muscle mass, is recognized to increase after stroke, although it can also be a pre-existing condition especially in the elder age. Both cases have been associated with a worse functional outcome (5, 6), highlighting the importance of measuring skeletal muscle mass as a measure of sarcopenia in a stroke population. However, the role of sarcopenia in ICH remains largely unclear.

The diagnostic tools to assess sarcopenia are based on methods that estimate muscle quantity comprise, among others, magnetic resonance imaging (MRI) and computed tomography (CT) scans of the lumbar muscles obtained on abdominal CT scans (7–9). Estimation by measuring temporalis muscle thickness (TMT) has become a favorable approach for sarcopenia measurement which could be easily, fast and reliable be performed in routine imaging scans. This has been shown in cerebellar ischemia and traumatic brain injury as well (10, 11). The aim of the current study was to investigate the association of sarcopenia and TMT with functional outcome in ICH at hospital discharge and 90 days follow-up.

## Methods

### Study design

**Figure 1:**
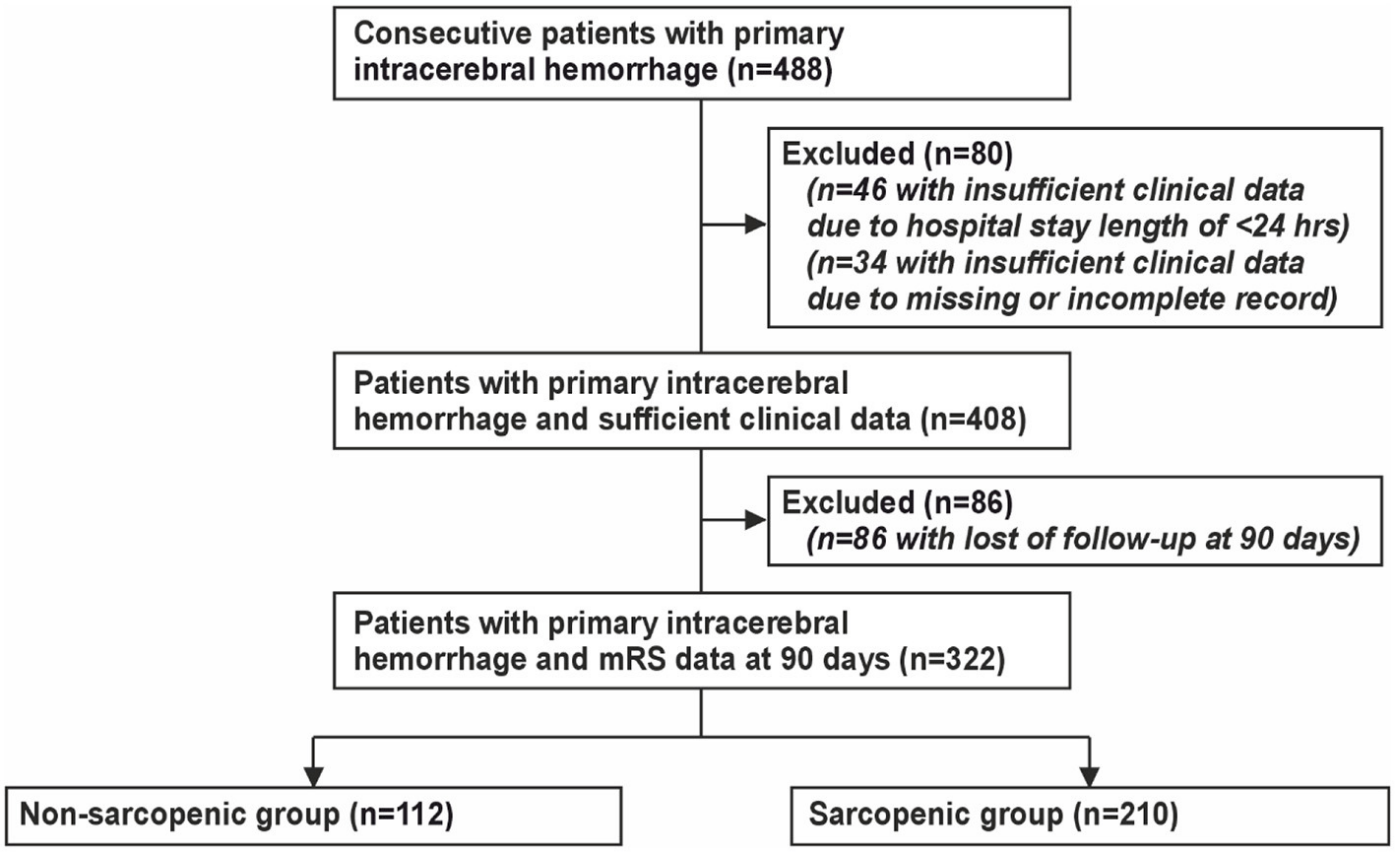
Study flowchart.

In this retrospective cross-sectional cohort study, we screened the hospital charts of 488 consecutive sICH patients admitted to the Department of Neurology of the University Medicine Rostock between January 2017 and December 2021. After exclusion of patients with insufficient clinical data due to hospital stay length < 24 h and patients with loss of follow-up at 90 days, a total of 322 patients were included in the final analysis (see Figure 1 for study flow chart). The study was approved by the Institutional Review Board of the Medical Faculty, University of Rostock (A-2017-0207).

### Patients

All patients received standard-of-care treatment according to the European Stroke Organization Guidelines for ICH (12). Basic characteristics like age, sex, body-mass-index (BMI), comorbidities (atrial hypertension, diabetes mellitus, atrial fibrillation, hyperlipoproteinemia, pneumonia) were obtained. Clinical severity of ICH was assessed using the modified ICH score (mICH) (13) and the National Institutes of Health Stroke Scale (NIHSS).

The topology of ICH was assessed by an experienced board-certified neuroradiologist (D.C.) blinded to the hypothesis investigated here by using cerebral CT or MRI. Since insular localization of ICH has been shown to influence functional outcome it was included in further analyses of candidate factors (14). Hemorrhage was considered to involve the insular region when at least a portion of the insula was compromised, regardless of affecting other brain regions. We determined whether there was insular involvement based on the identification of the appropriate ASPECTS region (15). The occurrence of intraventricular hemorrhage (IVH) was determined. ICH volume was estimated in cubic centimeters by the formula *ABC*/2 according to Kothari et al. (16) [see Wittstock and co-workers for details (14)]. The most likely cause of bleeding was determined considering the clinical and radiological characteristics and classified as follows: arterial hypertension, cerebral amyloid angiopathy according to the modified Boston criteria (17), hemorrhagic diathesis due to therapeutic anticoagulation.

Functional outcome [modified Rankin scale (mRS)] was assessed at hospital discharge as well as at 90 days follow-up, death rate was additionally determined at day 7.

### TMT measurement

TMT was assessed in CT or MRI scans at admission according to the method presented previously by Ravera et al. and Steindl et al. (18, 19). In detail, TMT was manually measured on the patient's baseline brain CT scan using the method introduced by Katsuki et al. (20). Slice thickness was set at 5 mm and the CT axial image was manually fixed to obtain a symmetric cut. TMT was measured bilaterally, perpendicular to the long axis of the temporal muscle. Three determinations were taken for each side: one at the level of the orbital roof, identified by comparing a sagittal cut, another at 5 mm above the orbital roof and the last at 5 mm below the orbital roof. The mean of the three measurements was calculated on both the left and the right side. Once the right and left means were obtained, the final TMT, expressed in millimeters, was measured by calculating the mean between the two values.

Based on the results, patients were further divided into two groups (sarcopenic vs. non-sarcopenic) as recommended by the European Working Group on Sarcopenia in Older People (EWGOP) and based on previously reported cut-off values (mean TMT 6.3 mm for male and 5.2 mm for female patients) (19, 21).

### Statistical analysis

Statistical comparisons between groups, Mann–Whitney *U*-test or Kruskal–Wallis test were used for comparison of parametric data, and Pearson Chi^2^-test or Fisher exact test for the comparison of non-parametric data as appropriate. For ordinal data, we used the Jonckheere–Terpstra test. *Post-hoc* analyses results were presented with Bonferroni adjustment. To test whether there was an association between categorical clinical variables and the outcome of interest, univariate and multivariate binary logistic (for in-hospital death as dependent outcome variable) or ordinal regression analyses (for mRS as dependent outcome measure) were performed. To select relevant covariates, we performed Mann–Whitney *U*-test and Chi^2^/Fisher Exact test in combination with univariate regression models to determine the predictive values and Odds ratios (OR) with 95% confidence intervals (95%CIs) of the candidate covariates age, sex, NIHSS at admission, ICH volume, intraventricular hemorrhage, mICH score, length of hospital stay, surgical intervention, pneumonia, and atrial fibrillation (see Supplementary Table S1). Prior to calculate multivariate regressions, assumptions of normality, homoscedasticity, independence of errors and absence of multicollinearity, were checked. For survival analyses, we performed univariate and multivariate Cox proportional hazards models to estimate hazard ratios (HRs) with 95%CIs and *P*-values for pairwise comparisons. The proportional hazards assumption was tested using log-log plots. Kaplan-Meier curve was used to visually compare the time to death after ICH onset in non-sarcopenic and sarcopenic patients.

Analyses were conducted using SPSS, version 25.0 (SPSS, Chicago, IL), and all *P*-values were two-sided and values of < 0.05 were deemed statistically significant (due to the limited sample size in our retrospective study, α adjusting of *P-*values was not carried out in order to preserve statistical power).

## Results

### Demographic and clinical characteristics

A total of 488 consecutive patients treated for ICH were screened, 80 were excluded (*n* = 46 due to hospital stay length of < 24 h and *n* = 34 due to insufficient clinical data). Another 86 patients were excluded due to loss of follow-up at 90 days (Figure 1). Out of 322 ICH patients (median age 77 years, IQR 66.0–83.0 years, 57.5% men), 294 patients (91.9%) showed supratentorial ICH, whereas 28 (8.1%) displayed infratentorial bleeding. Forty-three patients (27%) displayed insular involvement. Intraventricular hemorrhage occurred in 210 (65.2%) ICH patients. The median value of NIHSS at admission was 9 (IQR 4–15). Detailed demographic and clinical characteristics are illustrated in Table 1.

**Table 1:** Demographics, baseline clinical characteristics and management data of cohort with data at 90 days.

	**Total cohort (*n* = 322)**	**Non-sarcopenic group (*n* = 112)**	**Sarcopenic group (*n* = 210)**	***P*-value**
Males/females, *n* (%)	185 (57.5%)/137 (42.5%)	70 (62.5%)/42 (37.5%)	115 (54.8%)/95 (45.2%)	0.181^$^
Age (years), median (IQR)	77.0 (66.0–83.0)	67.5 (58.0–78.8)	80.0 (74.0–84.0)	**< 0.001** ^*^
BMI (kg/m^2^), median (IQR)	25.9 (23.5–29.1)	26.4 (24.2–30.2)	25.5 (23.2–28.3)	**0.025** ^*^
TMT (mm), median (IQR)	5.4 (4.7–6.2)	6.5 (5.5–6.9)	4.9 (4.4–5.6)	**< 0.001** ^*^
Sarcopenia, *n* (%)	210 (65.2%)	–	–	–
Length of hospital stay (days), median (IQR)	14 (9–20)	16 (10–22)	13 (8–20)	0.053^*^
NIHSS at admission, median (IQR)	9 (4–15)	8 (3–15)	9 (4–15)	0.708^*^
**Hemorrhage-related parameters**				
Modified ICH score, median (IQR)	4 (3–6)	3 (2–5)	4 (3–6)	**< 0.001** ^**#**^
ICH volume (ml), median (IQR)	14.6 (5.8–36.7)	15.4 (6.2–35.3)	14.0 (5.5–36.9)	0.751^*^
Supra-/infratentorial hemorrhage, *n* (%)	294 (91.9%)/28 (8.1%)	104 (92.9%)/8 (7.1%)	190 (90.5%)/20 (9.5%)	0.470^$^
Insular/non-insular hemorrhage, *n* (%)	79 (24.5%)/243 (75.5%)	31 (27.7%)/81 (72.3%)	48 (22.9%)/162 (77.1%)	0.338^$^
Intraventricular hemorrhage (IVH), *n* (%)	145 (45%)	52 (46.4%)	93 (44.3%)	0.713^$^
Accompanied SAH, *n* (%)	73 (22.7%)	23 (20.5%)	50 (23.8%)	0.504^$^
Surgical therapy, *n* (%)	19 (5.9%)	11 (9.8%)	8 (3.4%)	**0.029** ^**$**^
**Hemorrhage etiology**				
Anticoagulation associated, *n* (%)	78 (24.2%)	22 (19.6%)	56 (26.7%)	0.161^$^
Hypertension associated, *n* (%)	264 (82.0%)	90 (80.4%)	174 (82.9%)	0.578^$^
Amyloid angiopathy associated, *n* (%)	79 (24.5%)	25 (22.3%)	54 (25.7%)	0.500^$^
**Comorbidities**				
Arterial hypertension, *n* (%)	312 (96.9%)	104 (92.9%)	208 (99.0%)	**0.004** ^**$**^
Pneumonia, *n* (%)	139 (43.2%)	49 (43.8%)	90 (42.9%)	0.878^$^
Diabetes mellitus, *n* (%)	95 (29.5%)	32 (28.6%)	63 (30.0%)	0.789^$^
Hyperlipoproteinemia, *n* (%)	141 (43.8%)	46 (32.6%)	95 (45.2%)	0.473^$^
Treatment with statin, *n* (%)	109 (33.9%)	35 (31.3%)	74 (35.2%)	0.471^$^
Artrial fibrillation, *n* (%)	114 (35.4%)	28 (25.0%)	86 (41.0%)	**0.004** ^**$**^

Values are median and interquartile ranges (IQR, in brackets) or *n* (%). Significant *p*-values are provided as bold values.

ICH, Intracranial hemorrhage; BMI, body mass index; TMT, temporalis muscle thickness; NIHSS, National Institute of Health Stroke Scale; IVH, intraventricular hemorrhage; SAH, subarachnoidal hemorrhage; mRS, modified ranking scale.

^*^Mann-Whitney *U*-test.

^$^Pearson Chi^2^ or Fisher exact test as appropriate.

^#^Jonckheere-Terpstra test.

### Association of TMT with demographic and clinical characteristics

TMT values measured in the study cohort were significant higher in male than in female subjects [median (IQR) value 5.8 (5.3–6.6) vs. 4.8 (4.1–5.4); *P* < 0.001]. Significant correlation (Spearman rho, *P*-value) could be found for age (−0.52, 95% CI: −0.43 to −0.59; *P* < 0.001) as well as for BMI (0.29, 95% CI: −0.17 to −0.41; *P* < 0.001). Further analysis did not reveal any correlations with atrial fibrillation, pneumonia, hypertension, diabetes hyperlipoproteinemia, statin use, therapy with TAH as well as oral anticoagulation (data not shown).

### Association of sarcopenia with demographic and clinical characteristics

The full cohort was divided into a sarcopenic (65.2% of patients) and a non-sarcopenic group (34.8% of patients) based on the previously reported cut-off values (Table 1) (19). Sarcopenic patients were significantly older than non-sarcopenic patients (Table 1) and the frequency of sarcopenia displayed a clear age-dependency ranging from 25% in patients of age group < 50 years over 39% in age group 50–59 years, 48% in age group 60–69 years and 68% in age group 70–79 years up to 84% in age group 80+ years (*P* < 0.001, Chi^2^ test). Concerning other demographic and clinical characteristics, sarcopenic patients had significant higher NIHSS at admission, greater ICH volumes and higher mICH scores as compared to non-sarcopenic patients. The frequency of hypertension and atrial fibrillation (AF) as well was higher in sarcopenic vs. non-sarcopenic patients (Table 1). There were neither differences regarding the etiology of sICH nor the rate of pneumonia between sarcopenic vs. non-sarcopenic patients (Table 1).

### Association of TMT with functional outcome and mortality in ICH

Univariate regression models showed that TMT was associated with all major outcome parameters (mRS, death) at hospital discharge and at 90 days, with patients with higher TMT values having a more favorable outcome (see Table 2 for respective ORs). In contrast, TMT was not associated with early mortality rate at 7 days after ICH onset (Table 2). However, adjustment of ORs using multivariate regression models with variables influencing the outcome showed no significant associations of TMT and functional outcome/death at 7 days after ICH onset, hospital discharge and 90 days follow-up (Table 2).

**Table 2:** Odds ratios for functional outcomes by TMT and by non-sarcopenic vs. sarcopenic patient group.

	**Outcome rate** ^**a**^			**Univariate logistic/ordinal regression** ^**c**^		**Multivariate logistic/ordinal regression** ^**d**^	
	**Non-sarcopenic (*****n*** = **112)**	**Sarcopenic (*****n*** = **210)**	***P*** **-value** ^b^	**Unadjusted odds ratio (95% CI)**	***P*** **-value**	**Adjusted odds ratio (95% CI)**	***P*** **-value**
**TMT (mm) as marker for sarcopenia**							
mRS at discharge				0.83 (0.70–0.99)	**0.033**	0.95 (0.73–1.24)	0.708
mRS 6 (death) at discharge				0.76 (0.60–0.96)	**0.021**	0.95 (0.60–1.53)	0.838
90-days mRS^§^				0.78 (0.65–0.92)	**0.004**	0.99 (0.76–1.29)	0.919
mRS 6 (death) at 7 days				0.83 (0.62–1.11)	0.201	0.93 (0.59–1.45)	0.741
mRS 6 (death) at 90 days				0.75 (0.61–0.94)	**0.012**	0.98 (0.64–1.49)	0.914
**Non-sarcopenic vs. sarcopenic group -sarcopenic vs. sarcopenic group**							
mRS at discharge, median (IQR)	4.0 (2.0–5,8)	4.0 (3.0–6.0)	0.236	1.29 (0.85–1.92)	0.236	1.15 (0.70–1.90)	0.577
mRS 6, (death) at discharge *n* (%)	28 (25.0%)	67 (31.9%)	0.196	1.41 (0.84–2.36)	0.197	1.28 (0.79–3,45)	0.347
90-days mRS^§^, median (IQR)	3.0 (2.0–6.0)	4.0 (2,0–6.0)	0.104	1.41 (1.07–2.12)	0.049	1.01 (0.61–1.67)	0,968
mRS 6 (death) at 7 days, *n* (%)	14 (12.5%)	33 (15.7%)	0.437	1.31 (0.67–2.56)	0.437	1.14 (0.45–2.90)	0.781
mRS 6 (death) at 90 days, *n* (%)	34 (30.4%)	76 (36.2%)	0.293	1.32 (0.81–2.15)	0.270	1.12 (0.76–4.21)	0.632

ICH, Intracranial hemorrhage; BMI, body mass index; TMT, temporalis muscle thickness; NIHSS, National Institute of Health Stroke Scale; IVH, intraventricular hemorrhage; SAH, subarachnoidal hemorrhage; mRS, modified ranking scale. Significant *p*-values are provided as bold values.

^a^Number and (%) for death rate and median (IQR) for mRS.

^b^*P*-values are from Pearson Chi^2^ tests (death rate) or Jonckheere-Terpstra tests (mRS).

^c^Odds ratios and *P*-values from univariate ordinal or binary logistic regression analyses. An odds ratio >1 for the continuous variable (TMT) indicates a higher risk at higher values of TMT, while an odds ratio >1 for dichotomous risk factor (non-sarcopenic vs. sarcoopenic group) indicates a higher risk in the sarcopenic group.

^d^Adjustment of odds ratios for relevant covariates were performed by multivariate ordinal or binary logistic regression with predictive variables from univariate analyses (covariates for data at hospital discharge: sex, age, NIHSS at admission, ICH volume, insular hemorrhage, intraventricular hemorrhage, accompanied subarachnoidal hemorrhage, surgical therapy, pneumonia, atrial fibrillation, anticoagulation, and hypertension; covariates for data at 90 days: sex, age, NIHSS at admission, ICH volume, insular hemorrhage, intraventricular hemorrhage, accompanied subarachnoidal hemorrhage, pneumonia, atrial fibrillation, anticoagulation, and hypertension; covariates for data at 7 days: age, NIHSS at admission, ICH volume, insular hemorrhage, intraventricular hemorrhage and pneumonia).

Notably, after exclusion of age as a covariate, which was correlated to TMT, common OR for TMT from multivariate logistic ordinal regression at hospital discharge was 0.75 (95% CI: 0.60–0.94; *P* = 0.012). Common OR for TMT from multivariate logistic ordinal regression at 90 days follow-up was 0.85 (95% CI: 0.68–1.06; *P* = 0.152).

### Association of sarcopenia with functional outcome and mortality in ICH

**Figure 2:**
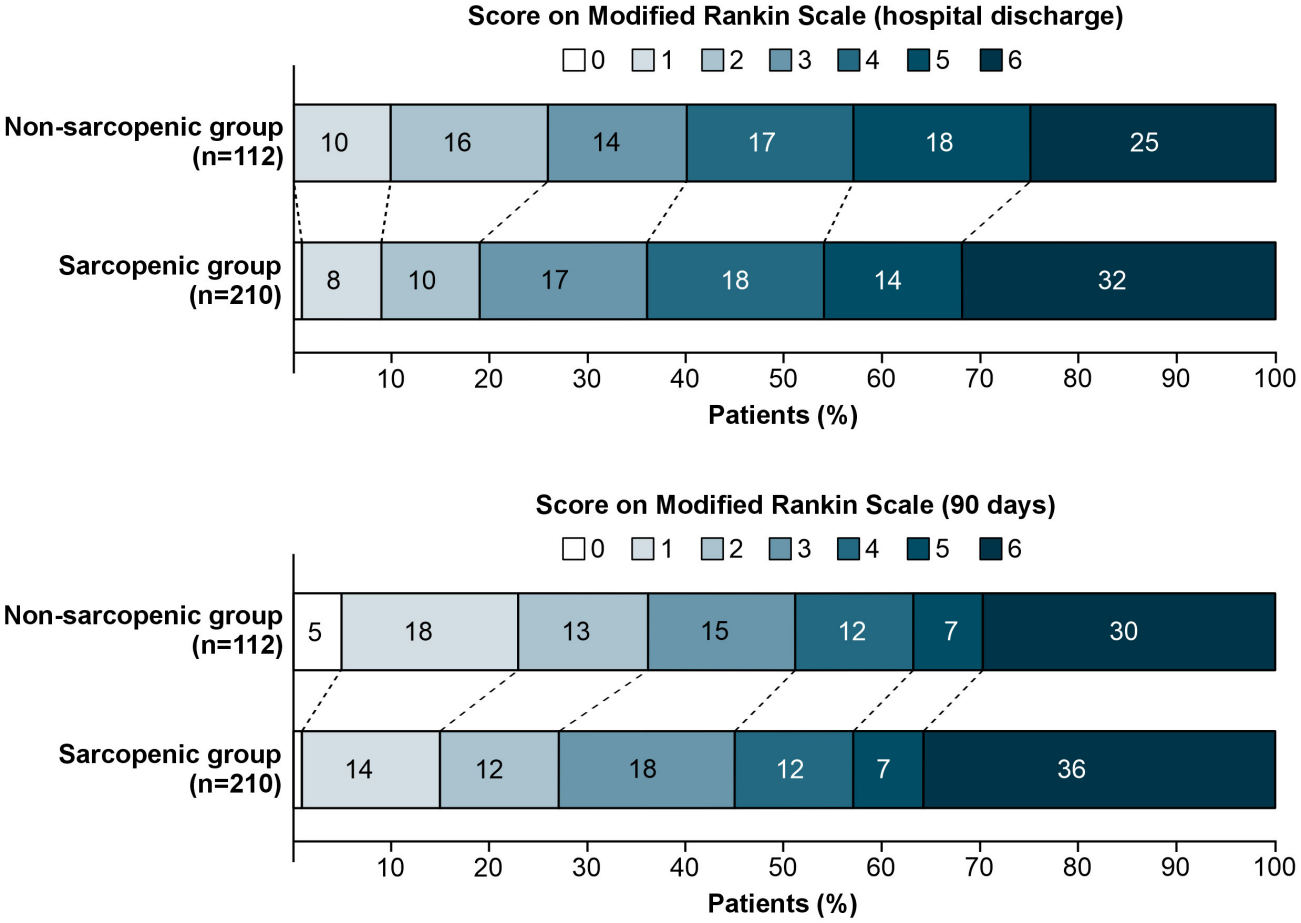
Modified rankin scale scores at discharge from hospital and at 90 days. Shown are the results of the ordinal analysis of the modified Rankin scale scores at discharge from hospital (upper panel) and at 90 days (lower panel). Scores range from 0 to 6, with 0 indicating no neurologic deficit, 1 no clinically significant disability, 2 slight disability (able to handle own affairs without assistance but unable to carry out all previous activities), 3 moderate disability requiring some help (e.g., with shopping, cleaning, and finances but able to walk unassisted), 4 moderately severe disability (unable to attend to bodily needs without assistance and unable to walk unassisted), 5 severe disability (requiring constant nursing care and attention), and 6 death. There was no significant difference between the non-sarcopenic and sarcopenic patient group at discharge (unadjusted common odds ratio: 1.28; 95% CI: 0.85–1.92; *P* = 0.236), but a significant difference at 90 days favoring the non-sarcopenic group over the sarcopenic group in the overall distribution of scores (unadjusted common odds ratio: 1.41; 95% CI: 1.07–2.12; *P* = 0.049).

As displayed in Figure 2, there was no significant difference in functional outcome between sarcopenic and non-sarcopenic patients at hospital discharge (unadjusted common OR: 1.28; 95% CI: 0.85–1.92; *P* = 0.236) and no difference in mortality rates at 7 days after ICH onset (unadjusted OR: 1.31; 95% CI: 0.67–2.56; *P* = 0.437) and at hospital discharge (unadjusted common OR: 1.41; 95% CI: 0.84–2.36; *P* = 0.197), but a significant difference at 90 days follow-up was observed favoring the non-sarcopenic group over the sarcopenic group (unadjusted common OR: 1.41; 95% CI: 1.07–2.12; *P* = 0.049). However, after adjustment of ORs using multivariate regression models with variables influencing the outcome revealed no significant associations of sarcopenia and functional outcome/death at 7 days after ICH onset, hospital discharge or 90 days follow-up (Table 2).

### Influence of other candidate risk factors on functional outcome and mortality in ICH

Univariate regression analyses revealed that several clinical candidate risk factors of unfavorable outcome such as sex, age, pneumonia as well as major ICH imaging parameters including ICH volume, intraventricular hemorrhage and insular localization were associated with functional outcome at hospital discharge and 90 days follow-up (see Supplementary Tables S1, S2). Multivariate regression analysis confirmed sex, age, NIHSS at admission, ICH volume, insular localization, intraventricular hemorrhage and pneumonia as significant predictors for functional outcome at hospital discharge (Supplementary Table S3). At 90 days follow-up, multivariate regression analysis showed associations of sex, age, NIHSS at admission, ICH volume, intraventricular hemorrhage and pneumonia with functional outcome (Supplementary Table S4). Early death rate at 7 days after ICH onset was mainly associated with age, ICH parameters and pneumonia (Supplementary Table S5).

### Influence of TMT and sarcopenia on survival after ICH

Univariate Cox regression analysis showed that TMT but not sarcopenia was significantly associated with survival after ICH, with patients with higher TMT values having a greater chance of survival [HR for TMT: 0.78 (95% CI: 0.65–0.94; *P* = 0.006); HR for sarcopenia: 1.38 (95%CI: 0.92–2.07; *P* = 0.124); Kaplan-Meier survival curve showing the association between sarcopenia and survival after ICH is displayed in Supplementary Figure S1]. However, adjustment of HRs using multivariate Cox regression models with variables influencing survival showed no significant associations of TMT and sarcopenia with survival (Supplementary Table S6).

Importantly, after exclusion of age as a covariate, which was correlated to TMT and sarcopenia, Cox regression analyses showed direct association of survival after ICH and TMT with a HR of 0.76 (95% CI: 0.61–0.94; *P* = 0.013) and increased survival for non-sarcopenic as compared to sarcopenic patients with a HR of 1.67 (95% CI: 1.08–2.58; *P* = 0.021).

## Discussion

The aim of the presented investigation was to elucidate the relationship between sarcopenia assessed by TMT measurement and ICH outcome. Since we did not detect relevant age-independent associations between TMT itself or sarcopenia with functional outcome, TMT seems to have limited prognostic value in ICH. Adjustment of ORs using multivariate regression models with variables influencing the outcome showed no significant associations of TMT and functional outcome/death at 7 days after ICH onset, hospital discharge and 90 days follow-up. Adjustment of HRs using multivariate Cox regression models with variables influencing survival showed no significant associations of TMT and sarcopenia with survival. However, other candidate factors affecting the prognosis of ICH such as sex, age, disease severity at admission, ICH volume, intraventricular hemorrhage, insular localization and pneumonia were confirmed to be associated with functional outcome or death after ICH.

Importantly, after exclusion of age as a covariate, which was correlated to TMT and sarcopenia, Cox regression analyses showed direct association of survival after ICH and TMT with a HR of 0.76 (95% CI: 0.61–0.94; *P* = 0.013) and increased survival for non-sarcopenic as compared to sarcopenic patients with a HR of 1.67 (95% CI: 1.08–2.58; *P* = 0.021).

In line with previous published findings, TMT was found to be higher in men than in women (22–24), likely related to the fact that males have more skeletal muscle mass than women both in absolute terms and related to BMI (25). The same studies showed a correlation with age, which was also confirmed in our study with younger patients having a higher TMT (26). Regarding the association of TMT with BMI, data from the literature show inconclusive results: in some studies a positive association between TMT and BMI has been described (6, 24), while in other reports no correlations were found (27). A positive correlation between BMI and TMT was identified in our ICH patient cohort. It is however important to note that BMI is a parameter based on weight and not on body shape, consequently, it does not allow the identification of sarcopenia particularly in obese patients (27). BMI did however not show any significant association with functional outcome.

The prevalence of sarcopenia is variable in the general population, probably due to the absence of uniform criteria (18). In our study, the percentage of patients with sarcopenia was found to be 65%, higher than in other studies mainly referring to populations of community-dwelling subjects (28–31). This could be due to different factors: Firstly, the median age of our study (77 years) is located in the upper range of related studies (29–32). Since sarcopenia is an age-related disorder, its prevalence is expected to be higher in the elderly population (33, 34). Indeed, we observed a clear age-dependency of sarcopenia frequency also in our cohort. Secondly, the assessment tools for diagnosing sarcopenia largely varied from clinical scoring to quantitative surrogate markers of muscular mass. Indeed, there is no common standard definition of sarcopenia available yet (18). Herein we used TMT as a quantitative surrogate marker of sarcopenia combined with previously generated cut-off values according to the European Working Group on Sarcopenia in Older People (EWGOP) (19, 21). Although the prevalence of sarcopenia in stroke populations may be variable with percentages ranging from 33 to 68% (33–35), our frequency of sarcopenia in an old ICH cohort is well in line with previous reports in similar cohorts.

Outcome prediction models relying on baseline variables can only partly forecast post-ICH outcome, and certain individuals—particularly elderly patients—may still face a lower likelihood of functional recovery (36, 37). In this scenario the investigation of new prognostic markers is of paramount importance, to maximize the benefit to patients while minimizing the harm in this frail population. In previous studies, the association between TMT or sarcopenia and functional outcome was mainly investigated in patients with stroke, subdural hematoma, traumatic brain injury and brain metastases (5, 6, 10, 11, 27, 38). In these studies, a negative correlation between TMT or premorbid sarcopenia and overall survival was demonstrated. In ischemic stroke patients several studies investigated the relationship of TMT/sarcopenia and overall survival with inconsistent results. Li et al. and Ravera et al. showed a negative correlation of TMT and functional outcome as well as overall survival (23, 33). On the other side, Lin et al. reported a positive correlation between TMT and a higher likelihood of functional independence measured by mRS at 90 day in an ischemic stroke cohort, who underwent endovascular therapy for large vessel occlusion without correlation with mortality (39).

In contrast, our data do not demonstrate any relevant age-independent associations between TMT itself or sarcopenia with functional outcome (mRS) or death at hospital discharge and at 90 days in ICH. The relatively high percentage of patients lost to follow-up at 90 days was due to local circumstances. The metropole region of Rostock is located in a wide-ranged rural landscape, has a relatively high mean age of residents and is a highly frequented touristic region.

Thus, sarcopenia as assessed by TMT seems to have only limited prognostic value in ICH. The reasons for this discrepancy might not only be related to different methods for assessing sarcopenia (clinical scoring vs. objective surrogate marker assessment) and study cohort characteristics such as age, but also in differences in temporal dynamics of the various brain disorders including variant recovery trajectories with ICH being associated with high early mortality and early functional disability burden (40–43). It could therefore be that the latency of 90 days until the measurement of functional outcome as used in the present study and as routinely applied in studies in acute brain diseases including ICH (42) is too short to detect the prognostic value of sarcopenia in ICH. Evaluation of cognition or physical fitness in geriatric or ICH patients is mainly based on cooperation, examination or anamnestic data from caregivers. TMT measurement could add an independent measure of sarcopenia in those patients. Existing prognostic scores in ICH such as the modified ICH score (which was used in our study), Functional Outcome in Patients with Primary Intracerebral Hemorrhage score (FUNC) or Modified Emergency Department Intracerebral Hemorrhage score (mEDICH) are not specifically validated in very old patients (44). A recent study (45) showed excellent capability of discriminating the group of elderly patients at risk of short-term death. Additional TMT measurement might add a structural surrogate of sarcopenia in those patients reflecting risk of short-term functional outcome.

Of note, we could confirm a variety of previously known prognostic factors like sex, age, NIHSS at admission, ICH volume, insular localization, intraventricular hemorrhage and pneumonia as substantially associated with functional outcome/death at hospital discharge and 90 days (14, 46).

The present study has several limitations. The main limitations derive from its retrospective nature and the monocentric cohort of patients enrolled. Therefore, e.g., a Charlson Comorbidity index as a compound measure of candidate factors impacting outcome could not be determined (47). However, major comorbidities with potential influence on functional outcome or death within 90 days after ICH onset were documented in patient records (see Table 1 for details). Our results are intended to guide further multicentric prospective validation studies on this topic. The retrospective study design is also conditional for the standard outcome measure latency of up to 90 days, which might be too short to detect relevant prognostic factors for the long-term outcome in ICH. However, already established prognostic factor in ICH were confirmed by the present study such sex, age, symptom severity at admission, several ICH parameters and pneumonia implicating a sufficient validity of our study. Another limit is that no officially accepted TMT cut-off value from literature for the definition of sarcopenia is available: we used the one obtained from the largest population studied and obtained according to current guidelines (19, 21), that also accounted for the difference existing between males and females. On the other hand, a strength of our approach is that our measurement protocol appears to be reliable and easily adoptable in the routine clinical practice, as takes only a few additional minutes and can be potentially automated.

Despite of confirmation of several known prognostic factors like ICH volume, insular localization, intraventricular hemorrhage and others, sarcopenia could not be detected among these. Reasons for this finding remained unclear; larger and prospective studies with longer observation periods are warranted.

## Data Availability

The raw data supporting the conclusions of this article will be made available by the authors, without undue reservation.

## References

[1] Keep RF Hua Y Xi G. Intracerebral haemorrhage: mechanisms of injury and therapeutic targets. Lancet Neurol. (2012) 11:720–31. 10.1016/s1474-4422(12)70104-7 22698888 PMC3884550

[2] Gerner ST Kuramatsu JB Moeller S Huber A Lücking H Kloska SP. Specific lobar affection reveals a rostrocaudal gradient in functional outcome in spontaneous intracerebral hemorrhage. Stroke. (2017) 48:587–95. 10.1161/strokeaha.116.015890 28179560

[3] Kuramatsu JB Huttner HB Schwab S. Advances in the management of intracerebral hemorrhage. J Neur Trans. (2013) 120(Suppl. 1):S35–41. 10.1007/s00702-013-1040-y 23720189

[4] van Asch CJ Luitse MJ Rinkel GJ van der Tweel I Algra A Klijn CJ. Incidence, case fatality, and functional outcome of intracerebral haemorrhage over time, according to age, sex, and ethnic origin: a systematic review and meta-analysis. Lancet Neurol. (2010) 9:167–76. 10.1016/s1474-4422(09)70340-0 20056489

[5] Nozoe M Kanai M Kubo H Yamamoto M Shimada S Mase K. Prestroke sarcopenia and functional outcomes in elderly patients who have had an acute stroke: A prospective cohort study. Nutrition. (2019) 66:44–7. 10.1016/j.nut.2019.04.011 31207438

[6] Ohyama K Watanabe M Nosaki Y Hara T Iwai K Mokuno K. Correlation between skeletal muscle mass deficit and poor functional outcome in patients with acute ischemic stroke. J Stroke Cerebrovasc Dis. (2020) 29:104623. 10.1016/j.jstrokecerebrovasdis.2019.104623 31952978

[7] Beaudart C McCloskey E Bruyère O Cesari M Rolland Y Rizzoli R. Sarcopenia in daily practice: assessment and management. BMC Geriatr. (2016) 16:170. 10.1186/s12877-016-0349-4 27716195 PMC5052976

[8] Buckinx F Landi F Cesari M Fielding RA Visser M Engelke K. Pitfalls in the measurement of muscle mass: a need for a reference standard. J Cachexia Sarcopenia Muscle. (2018) 9:269–78. 10.1002/jcsm.12268 29349935 PMC5879987

[9] MasanésFRojanoILXSalvàASerra-RexachJAArtazaIFormigaF. Cut-off points for muscle mass - not grip strength or gait speed - determine variations in sarcopenia prevalence. J Nutr Health Aging. (2017) 21:825–9. 10.1007/s12603-016-0844-528717813

[10] Dubinski D Won SY Mattes I Trnovec S Behmanesh B Cantré D. Frailty in cerebellar ischemic stroke-The significance of temporal muscle thickness. Front Neurol. (2023) 14:1193685. 10.3389/fneur.2023.1193685 37822528 PMC10562580

[11] Dubinski D Won SY Meyer-Wilmes J Trnovec S Rafaelian A Behmanesh B. Frailty in traumatic brain injury-the significance of temporal muscle thickness. J Clin Med. (2023) 12:7625. 10.3390/jcm12247625 38137693 PMC10743381

[12] Steiner T Al-Shahi Salman R Beer R Christensen H Cordonnier C Csiba L. European Stroke Organisation (ESO) guidelines for the management of spontaneous intracerebral hemorrhage. Int J Stroke. (2014) 9:840–55. 10.1111/ijs.12309 25156220

[13] Godoy DA Piñero G Di Napoli M. Predicting mortality in spontaneous intracerebral hemorrhage: can modification to original score improve the prediction? Stroke. (2006) 37:1038–44. 10.1161/01.Str.0000206441.79646.49 16514104

[14] Wittstock M Meyer K Klinke J Grossmann A Walter U Storch A. Effects of insular involvement on functional outcome after intracerebral hemorrhage. Acta Neurol Scand. (2021) 144:559–65. 10.1111/ane.13496 34224142

[15] Barber PA Demchuk AM Zhang J Buchan AM. Validity and reliability of a quantitative computed tomography score in predicting outcome of hyperacute stroke before thrombolytic therapy. ASPECTS Study Group Alberta Stroke Programme Early CT Score. Lancet. (2000) 355:1670–4. 10.1016/s0140-6736(00)02237-6 10905241

[16] Kothari RU Brott T Broderick JP Barsan WG Sauerbeck LR Zuccarello M. The ABCs of measuring intracerebral hemorrhage volumes. Stroke. (1996) 27:1304–5. 10.1161/01.str.27.8.1304 8711791

[17] Linn J Halpin A Demaerel P Ruhland J Giese AD Dichgans M. Prevalence of superficial siderosis in patients with cerebral amyloid angiopathy. Neurology. (2010) 74:1346–50. 10.1212/WNL.0b013e3181dad605 20421578 PMC2875936

[18] Dhillon RJ Hasni S. Pathogenesis and management of sarcopenia. Clin Geriatr Med. (2017) 33:17–26. 10.1016/j.cger.2016.08.002 27886695 PMC5127276

[19] Steindl A Leitner J Schwarz M Nenning KH Asenbaum U Mayer S. Sarcopenia in neurological patients: standard values for temporal muscle thickness and muscle strength evaluation. J Clin Med. (2020) 9:51272. 10.3390/jcm9051272 32354003 PMC7288067

[20] Katsuki M Kakizawa Y Nishikawa A Yamamoto Y Uchiyama T Agata M. temporal muscle and stroke-a narrative review on current meaning and clinical applications of temporal muscle thickness, area, and volume. Nutrients. (2022) 14:687. 10.3390/nu14030687 35277046 PMC8840759

[21] Cruz-Jentoft AJ Bahat G Bauer J Boirie Y Bruyère O Cederholm T. Sarcopenia: revised European consensus on definition and diagnosis. Age Ageing. (2019) 48:16–31. 10.1093/ageing/afy169 30312372 PMC6322506

[22] An G Ahn S Park JS Jeun SS Hong YK. Association between temporal muscle thickness and clinical outcomes in patients with newly diagnosed glioblastoma. J Cancer Res Clin Oncol. (2021) 147:901–9. 10.1007/s00432-020-03386-5 32929611 PMC11802135

[23] Li YX Hou J Liu WY. Long-term prognostic significance of sarcopenia in acute ischemic stroke. Medicine. (2022) 101:e30031. 10.1097/md.0000000000030031 36042682 PMC9410603

[24] Nozoe M Kubo H Kanai M Yamamoto M Okakita M Suzuki H. Reliability and validity of measuring temporal muscle thickness as the evaluation of sarcopenia risk and the relationship with functional outcome in older patients with acute stroke. Clin Neurol Neurosurg. (2021) 201:106444. 10.1016/j.clineuro.2020.106444 33395619

[25] Tay L Ding YY Leung BP Ismail NH Yeo A Yew S. Sex-specific differences in risk factors for sarcopenia amongst community-dwelling older adults. Age. (2015) 37:121. 10.1007/s11357-015-9860-3 26607157 PMC5005859

[26] Chen LK Woo J Assantachai P Auyeung TW Chou MY Iijima K. Asian Working Group for Sarcopenia: 2019 Consensus Update on Sarcopenia Diagnosis and Treatment. J Am Med Direct Assoc. (2020) 21:300–7.e302. 10.1016/j.jamda.2019.12.012 32033882

[27] Furtner J Weller M Weber M Gorlia T Nabors B Reardon DA. Temporal muscle thickness as a prognostic marker in patients with newly diagnosed glioblastoma: translational imaging analysis of the CENTRIC EORTC 26071-22072 and CORE Trials. Clin Cancer Res. (2022) 28:129–36. 10.1158/1078-0432.Ccr-21-1987 34667022

[28] Cruz-Jentoft AJ Landi F Schneider SM Zúñiga C Arai H Boirie Y. Prevalence of and interventions for sarcopenia in ageing adults: a systematic review. Report of the International Sarcopenia Initiative (EWGSOP and IWGS). Age Ageing. (2014) 43:748–59. 10.1093/ageing/afu115 25241753 PMC4204661

[29] Gao Q Hu K Yan C Zhao B Mei F Chen F. Associated factors of sarcopenia in community-dwelling older adults: a systematic review and meta-analysis. Nutrients. (2021) 13:4291. 10.3390/nu13124291 34959843 PMC8707132

[30] Petermann-Rocha F Balntzi V Gray SR Lara J Ho FK Pell JP. Global prevalence of sarcopenia and severe sarcopenia: a systematic review and meta-analysis. J Cachexia Sarcopenia Muscle. (2022) 13:86–99. 10.1002/jcsm.12783 34816624 PMC8818604

[31] Su Y Yuki M Otsuki M. Prevalence of stroke-related sarcopenia: a systematic review and meta-analysis. J Stroke Cerebrovasc Dis. (2020) 29:105092. 10.1016/j.jstrokecerebrovasdis.2020.105092 32807486

[32] Xu J Wan CS Ktoris K Reijnierse EM Maier AB. Sarcopenia is associated with mortality in adults: a systematic review and meta-analysis. Gerontology. (2022) 68:361–76. 10.1159/000517099 34315158

[33] Ravera B Lombardi C Bellavia S Scala I Cerulli F Torchia E. Temporalis muscle thickness as a predictor of functional outcome after reperfusion therapies for acute ischemic stroke: a retrospective, cohort study. J Neurol. (2024) 271:6015–24. 10.1007/s00415-024-12575-y 39028361

[34] Kim YH Choi YA. Prevalence and risk factors of possible sarcopenia in patients with subacute stroke. PLoS ONE. (2023) 18:e0291452. 10.1371/journal.pone.0291452 37725595 PMC10508606

[35] Ikeji R Nozoe M Yamamoto M Seike H Kubo H Shimada S. Sarcopenia in patients following stroke: prevalence and associated factors. Clin Neurol Neurosurg. (2023) 233:107910. 10.1016/j.clineuro.2023.107910 37531752

[36] Alawieh A Chatterjee A Feng W Porto G Vargas J Kellogg R. Thrombectomy for acute ischemic stroke in the elderly: a 'real world' experience. J Neurointerv Surg. (2018) 10:1209–17. 10.1136/neurintsurg-2018-013787 29666180

[37] Meyer L Alexandrou M Flottmann F Deb-Chatterji M Abdullayev N Maus V. Endovascular Treatment of Very Elderly Patients Aged ≥90 With Acute Ischemic Stroke. J Am Heart Assoc. (2020) 9:e014447. 10.1161/jaha.119.014447 32089059 PMC7335589

[38] Dubinski D Won SY Behmanesh B Cantré D Mattes I Trnovec S. Significance of temporal muscle thickness in chronic subdural hematoma. J Clin Med. (2022) 11:6456. 10.3390/jcm11216456 36362682 PMC9654786

[39] Lin YH Chung CT Chen CH Cheng CJ Chu HJ Chen KW. Association of temporalis muscle thickness with functional outcomes in patients undergoing endovascular thrombectomy. Eur J Radiol. (2023) 163:110808. 10.1016/j.ejrad.2023.110808 37080063

[40] Carhuapoma L Murthy S Shah VA. Outcome trajectories after intracerebral hemorrhage. Semin Neurol. (2024) 44:298–307. 10.1055/s-0044-1787104 38788763

[41] Puy L Parry-Jones AR Sandset EC Dowlatshahi D Ziai W Cordonnier C. Intracerebral haemorrhage. Nat Rev Dis Prim. (2023) 9:14. 10.1038/s41572-023-00424-7 36928219

[42] Massicotte S Lun R Yogendrakumar V Dewar B Chung HS Konder R. How outcomes are measured after spontaneous intracerebral hemorrhage: a systematic scoping review. PLoS ONE. (2021) 16:e0253964. 10.1371/journal.pone.0253964 34191862 PMC8244847

[43] Wei JW Heeley EL Wang JG Huang Y Wong LK Li Z. Comparison of recovery patterns and prognostic indicators for ischemic and hemorrhagic stroke in China: the ChinaQUEST (QUality Evaluation of Stroke Care and Treatment) Registry study. Stroke. (2010) 41:1877–83. 10.1161/strokeaha.110.586909 20651267

[44] Gregório T Pipa S Cavaleiro P Atanásio G Albuquerque I Chaves PC. Assessment and comparison of the four most extensively validated prognostic scales for intracerebral hemorrhage: systematic review with meta-analysis. Neurocrit Care. (2019) 30:449–66. 10.1007/s12028-018-0633-6 30426449

[45] Batista R PM Catamo Vaz D Buque H Nzwalo H Marreiros. Prognostic accuracy of common mortality prognostic scales in very old patients with intracerebral haemorrhge. Ann Neuroscience. (2023) 1–6. 10.1177/09727531231185200 PMC12267898 40688404

[46] Masotti L Grifoni E Migli L Dei A Spina R Calamai I. Prognostic determinants in patients with non traumatic intracerebral hemorrhage: a real life report. Acta Clin Belgica. (2021) 76:365–72. 10.1080/17843286.2020.1750151 32279610

[47] Bar B Hemphill JC 3rd. Charlson comorbidity index adjustment in intracerebral hemorrhage. Stroke. (2011) 42:2944–6. 10.1161/strokeaha.111.617639 21799172 PMC3183144

