# Supplementary material for: Prognostic value of temporalis muscle thickness as a marker of sarcopenia in intracerebral hemorrhage
Source: Front Neurol. 2025 May 16;16:1564550. doi: 10.3389/fneur.2025.1564550 (PMC12122341; doi:10.3389/fneur.2025.1564550)
Supplement: Supplementary file 1 [file Supplementary_file_1.docx]

**Prognostic value of temporalis muscle thickness in intracerebral hemorrhage**

**Supplementary Material**

**Supplementary Table S1:** Univariate statistical analyses of individual risk factors for functional outcomes at hospital discharge.

**Supplementary Table S2:** Univariate statistical analyses of individual risk factors for functional outcomes at 90 days.

**Supplementary Table S3:** Multivariate statistical analyses of risk factors for functional outcomes at hospital discharge.

**Supplementary Table S4:** Multivariate statistical analyses of risk factors for functional outcomes at 90 days.

**Supplementary Table S5.** Univariate and multivariate statistical analyses of risk factors for death within 7 days.

**Supplementary Table S6.** Univariate and multivariate Cox regression analyses of candidate risk factors for survival.

**Supplementary Figure S1.** Kaplan-Meier curve showing the association between sarcopenia and survival after intracerebral hemorrhage (ICH).

**SUPPLEMENTARY TABLE S1.** Univariate statistical analyses of individual risk factors for functional outcomes at hospital discharge.

|  | **mRS^a^** | |  | **mRS 0-1^b^** | |  | | **mRS 0-3^b^** | | |  | | **mRS 6 (in-hospital death)^b^** | | |  |
| --- | --- | --- | --- | --- | --- | --- | --- | --- | --- | --- | --- | --- | --- | --- | --- | --- |
|  | **Common odds ratio (95% CI)** | ***P* value** |  | **Odds ratio (95% CI)** | ***P* value** | |  | | **Odds ratio (95% CI)** | ***P* value** | |  | | **Odds ratio (95% CI)** | ***P* value** | |
| **Male/female** | 0.63 (0.43-0.94) | **0.022** |  | 1.72 (0.76-3.91) | 0.193 | |  | | 1.56 (0.98-2.48) | 0.061 | |  | | 0.56 (0.35-0.91) | **0.018** | |
| **Age (years)** | 1.03 (1.02-1.05) | **<0.001** |  | 0.95 (0.92-0.97) | **<0.001** | |  | | 0.98 (0.96-0.99) | **0.016** | |  | | 1.04 (1.02-1.07) | **<0.001** | |
| **BMI** | 1.00 (0.96-1.04) | 0.922 |  | 0.99 (0.92-1.06) | 0.704 | |  | | 0.99 (0.94-1.03) | 0.561 | |  | | 0.96 (0.91-1.02) | 0.181 | |
| **TMT (mm)** | 0.83 (0.70-0.99) | **0.033** |  | 1.43 (1.05-1.93) | **0.023** | |  | | 1.17 (1.02-1.43) | **0.022** | |  | | 0.76 (0.60-0.96) | **0.021** | |
| **Sarcopenia** | 1.29 (0.85-1.92) | 0.236 |  | 0.86 (0.39-1.89) | 0.709 | |  | | 0.83 (0.52-1.33) | 0.430 | |  | | 1.41 (0.84-2.36) | 0.197 | |
| **NIHSS at admission** | 1.30 (1.25-1.35) | **<0.001** |  | 0.67 (0.56-0.79) | **<0.001** | |  | | 0.73 (0.68-0.79) | **<0.001** | |  | | 1.21 (1.15-1.26) | **<0.001** | |
| **ICH volume (ml)** | 1.04 (1.03-1.04) | **<0.001** |  | 0.94 (0.90-0.98) | **0.003** | |  | | 0.95 (0.93-0.96) | **<0.001** | |  | | 1.03 (1.02-1.04) | **<0.001** | |
| **Insular hemorrhage** | 4.58 (2.80-7.55) | **<0.001** |  | 0.21 (0.05-0.90) | **0.035** | |  | | 0.18 (0.09-0.36) | **<0.001** | |  | | 4.07 (2.38-6.96) | **<0.001** | |
| **Intraventricular hemorrhage (IVH)** | 10.03 (6.34-15.86) | **<0.001** |  | 0.12 (0.04-0.41) | **<0.001** | |  | | 0.07 (0.04-0.13) | **<0.001** | |  | | 9.16 (5.15-16.31) | **<0.001** | |
| **Accompanied SAH** | 2.02 (1.26-3.24) | **0.004** |  | 0.88 (0.34-2.25) | 0.789 | |  | | 0.47 (0.26-0.85) | **0.012** | |  | | 1.98 (1.15-3.41) | **0.014** | |
| **Surgical therapy** | 2.90 (1.21-6.94) | **0.017** |  | - | - | |  | | - | **-** | |  | | 1.81 (0.70-4.64) | 0.220 | |
| **Modified ICH score** | 2.45 (2.14-2.80) | **<0.001** |  | 0.41 (0.30-0.56) | **<0.001** | |  | | 0.40 (0.32-0.49) | **<0.001** | |  | | 2.42 (1.99-2.94) | **<0.001** | |
| **Pneumonia** | 19.55 (11.72-32.55) | **<0.001** |  | 0.08 (0.02-0.36) | **<0.001** | |  | | 0.07 (0.03-0.13) | **<0.001** | |  | | 21.12 (10.74-41.54) | **<0.001** | |
| **Atrial fibrillation** | 2.70 (1.74-4.19) | **<0.001** |  | 0.25 (0.07-0.84) | **0.025** | |  | | 0.42 (0.25-0.72) | **0.002** | |  | | 2.72 (1.63-4.51) | **<0.001** | |
| **Anticoagulation** | 2.38 (1.49-4.74) | **<0.001** |  | 0.13 (0.04-0.49) | **0.003** | |  | | 0.14 (0.03-0.67) | **0.014** | |  | | 2.50 (1.47-4.25) | **<0.001** | |
| **Hypertension** | 9.30 (2.89-29.93) | **<0.001** |  | 0.18 (0.05-0.61) | **0.006** | |  | | 0.10 (0.02-0.45) | **0.003** | |  | | - | - | |
| **Diabetes mellitus** | 1.11 (0.72-1.69) | 0.635 |  | 1.52 (0.69-3.36) | 0.300 | |  | | 0.91 (0.56-1.50) | 0.723 | |  | | 1.07 (0.64-1.81) | 0.795 | |

BMI: body mass index; ICH: intracranial hemorrhage; IVH: intraventricular hemorrhage; mRS: modified ranking scale; NIHSS: National Institute of Health Stroke Scale; SAH: subarachnoidal hemorrhage; TMT: temporalis muscle thickness.

^a^Odds ratios and *P* values from univariate logistic ordinal regression analyses. An Odds ratio >1 for sex as a risk factor indicates a higher risk for the outcome/death in men, an Odds ratio >1 for dichotomous risk factors indicates a higher risk in the presence of the respective risk factor, while an Odds ratio >1 for continuous variables indicates a higher risk at higher values of the potential risk factor.

^b^Odds ratios and *P* values from univariate logistic binary regression analyses. An Odds ratio >1 for sex as a risk factor indicates a higher risk for the outcome/death in men, an Odds ratio >1 for dichotomous risk factors indicates a higher risk in the presence of the respective risk factor, while an Odds ratio >1 for continuous variables indicates a higher risk at higher values of the potential risk factor.

**SUPPLEMENTARY TABLE S2.** Univariate statistical analyses of individual risk factors for functional outcomes at 90 days.

|  | **90-days mRS^a^** | |  | **90-days mRS 0-1^b^** | |  | | **90-days mRS 0-3^b^** | | |  | | **90-days mRS 6 (death)^b^** | | |
| --- | --- | --- | --- | --- | --- | --- | --- | --- | --- | --- | --- | --- | --- | --- | --- |
|  | **Common odds ratio (95% CI)** | ***P* value** |  | **Odds ratio (95% CI)** | ***P* value** | |  | | **Odds ratio (95% CI)** | ***P* value** | |  | | **Odds ratio (95% CI)** | ***P* value** |
| **Male/female** | 0.61 (0.41-0.91) | **0.016** |  | 1.24 (0.69-2.26) | 0.473 | |  | | 1.61 (1.03-2.52) | **0.037** | |  | | 0.52 (0.33-0.83) | **0.006** |
| **Age (years)** | 1.05 (1.03-1.06) | **<0.001** |  | 0.95 (0.93-0.97) | **<0.001** | |  | | 0.96 (0.94-0.98) | **<0.001** | |  | | 1.04 (1.02-1.07) | **<0.001** |
| **BMI** | 1.00 (0.96-1.04) | 0.838 |  | 0.96 (0.91-1.03) | 0.240 | |  | | 1.01 (0.97-1.06) | 0.628 | |  | | 0.96 (0.90-1.01) | 0.113 |
| **TMT (mm)** | 0.78 (0.65-0.92) | **0.004** |  | 1.39 (1.09-1.77) | **0.009** | |  | | 1.26 (1.03-1.54) | **0.023** | |  | | 0.75 (0.61-0.94) | **0.012** |
| **Sarcopenia** | 1.41 (1.07-2.12) | **0.049** |  | 0.58 (0.32-1.05) | 0.070 | |  | | 0.78 (0.49-1.24) | 0.294 | |  | | 1.32 (0.81-2.15) | 0.270 |
| **NIHSS at admission** | 1.23 (1.19-1.28) | **<0.001** |  | 0.75 (0.68-0.82) | **<0.001** | |  | | 0.80 (0.76-0.85) | **<0.001** | |  | | 1.18 (1.1541.24) | **<0.001** |
| **ICH volume (ml)** | 1.03 (1.02-1.04) | **<0.001** |  | 0.96 (0.94-0.98) | **<0.001** | |  | | 0.97 (0.95-0.98) | **<0.001** | |  | | 1.03 (1.02-1.04) | **<0.001** |
| **Insular hemorrhage** | 3.20 (1.98-5.18) | **<0.001** |  | 0.40 (0.17-0.91) | **0.030** | |  | | 0.34 (0.20-0.59) | **<0.001** | |  | | 3.45 (2.04-5.83) | **<0.001** |
| **Intraventricular hemorrhage (IVH)** | 7.73 (4.95-12.06) | **<0.001** |  | 0.03 (0.01-0.14) | **<0.001** | |  | | 0.12 (0.07-0.20) | **<0.001** | |  | | 6.11 (3.69-10.18) | **<0.001** |
| **Accompanied SAH** | 2.34 (1.44-3.78) | **<0.001** |  | 0.53 (0.24-1.18) | 0.119 | |  | | 0.44 (0.25-0.76) | **0.003** | |  | | 2.44 (1.43-4.17) | **0.001** |
| **Surgical therapy** | 2.27 (0.95-5.41) | 0.065 |  | - | - | |  | | 0.28 (0.09-0.87) | **0.028** | |  | | 1.78 (0.70-4.52) | 0.224 |
| **Modified ICH score** | 2.32 (2.00-2.60) | **<0.001** |  | 0.42 (0.33-0.54) | **<0.001** | |  | | 0.40 (0.33-0.49) | **<0.001** | |  | | 2.13 (1.80-2.51) | **<0.001** |
| **Pneumonia** | 20.09 (12.05-33.52) | **<0.001** |  | 0.04 (0.01-0.15) | **<0.001** | |  | | 0.05 (0.03-0.09) | **<0.001** | |  | | 17.00 (9.42-30.69) | **<0.001** |
| **Atrial fibrillation** | 3.01 (1.92-4.71) | **<0.001** |  | 0.19 (0.08-0.50) | **<0.001** | |  | | 0.32 (0.19-0.54) | **<0.001** | |  | | 2.70 (1.65-4.42) | **<0.001** |
| **Anticoagulation** | 1.42 (1.02-6.76) | **0.034** |  | 0.68 (0.47-0.99) | **0.047** | |  | | 0.76 (0.62-0.94) | **0.013** | |  | | 1.35 (1.10-1.65) | **0.003** |
| **Hypertension** | 8.08 (2.50-26.10) | **<0.001** |  | 0.12 (0.03-0.46) | **0.002** | |  | | 0.09 (0.01-0.74) | **0.025** | |  | | - | - |
| **Diabetes mellitus** | 1.17 (0.76-1.79) | 0.469 |  | 0.98 (0.52-1.85) | 0.941 | |  | | 0.72 (0.44-1.16) | 0.175 | |  | | 0.90 (0.54-1.49) | 0.672 |

BMI: body mass index; ICH: intracranial hemorrhage; IVH: intraventricular hemorrhage; mRS: modified ranking scale; NIHSS: National Institute of Health Stroke Scale; SAH: subarachnoidal hemorrhage; TMT: temporalis muscle thickness.

^a^Odds ratios and *P* values from univariate ordinal regression analyses. An Odds ratio >1 for sex as a risk factor indicates a higher risk for the outcome/death in men, an Odds ratio >1 for dichotomous risk factors indicates a higher risk in the presence of the respective risk factor, while an Odds ratio >1 for continuous variables indicates a higher risk at higher values of the potential risk factor.

^b^Odds ratios and *P* values from univariate binary logistic regression analyses. An Odds ratio >1 for sex as a risk factor indicates a higher risk for the outcome/death in men, an Odds ratio >1 for dichotomous risk factors indicates a higher risk in the presence of the respective risk factor, while an Odds ratio >1 for continuous variables indicates a higher risk at higher values of the potential risk factor.

**SUPPLEMENTARY TABLE S3.** Multivariate statistical analyses of risk factors for functional outcomes at hospital discharge.

|  | **mRS^a^** | |  | **mRS 6 (in-hospital death)^b^** | |
| --- | --- | --- | --- | --- | --- |
|  | **Common odds ratio (95% CI)** | ***P* value** |  | **Odds ratio (95% CI)** | ***P* value** |
| **Male/female** | 0.48 (0.29-0.81) | **0.006** |  | 0.37 (0.15-0.90) | **0.028** |
| **Age (years)** | 1.04 (1.01-1.07) | **0.002** |  | 1.06 (1.01-1.11) | **0.016** |
| **TMT (mm)** | 0.95 (0.73-1.24) | 0.708 |  | 0.95 (0.60-1.53) | 0.838 |
| **NIHSS at admission** | 1.18 (1.13-1.24) | **<0.001** |  | 1.11 (1.05-1.18) | **<0.001** |
| **ICH volume (ml)** | 1.01 (1.00-1.02) | **0.032** |  | 1.02 (1.00-1.03) | **0.008** |
| **Insular hemorrhage** | 2.14 (1.17-3.19) | **0.013** |  | 1.70 (0.72-4.05) | 0.227 |
| **Intraventricular hemorrhage (IVH)** | 4.08 (2.36-7.06) | **<0.001** |  | 3.59 (1.49-8.64) | **0.004** |
| **Accompanied SAH** | 1.09 (0.62-1.93) | 0.766 |  | 0.91 (0.38-2.15) | 0.820 |
| **Surgical therapy** | 1.85 (0.67-5.12) | 0.239 |  | 0.58 (0.15-2.20) | 0.421 |
| **Pneumonia** | 5.29 (2.96-9.45) | **<0.001** |  | 9.69 (3.93-23.88) | **<0.001** |
| **Atrial fibrillation** | 1.05 (0.56-1.98) | 0.869 |  | 1.39 (0.54-3.53) | 0.495 |
| **Anticoagulation** | 1.40 (0.70-2.78) | 0.338 |  | 1.83 (0.65-5.14) | 0.252 |
| **Hypertension** | 1.23 (0.34-4.44) | 0.747 |  | - | - |

ICH: intracranial hemorrhage; IVH: intraventricular hemorrhage; mRS: modified ranking scale; NIHSS: National Institute of Health Stroke Scale; SAH: subarachnoidal hemorrhage; TMT: temporalis muscle thickness.

ICH: Intracranial hemorrhage TMT: temporalis muscle thickness; IVH: intraventricular hemorrhage; NIHSS: National Institute of Health Stroke Scale; mRS: modified ranking scale; TMT: temporalis muscle thickness.

^a^Odds ratios and *P* values from multivariate ordinal regression analyses using the variables resulted statistically significant in the univariate comparison as predictors. An Odds ratio >1 for sex as a risk factor indicates a higher risk for the outcome/death in men, an Odds ratio >1 for dichotomous risk factors indicates a higher risk in the presence of the respective risk factor, while an Odds ratio >1 for continuous variables indicates a higher risk at higher values of the potential risk factor.

^b^Odds ratios and *P* values from multivariate binary logistic regression analyses using the variables resulted statistically significant in the univariate comparison as predictors. An Odds ratio >1 for sex as a risk factor indicates a higher risk for the outcome/death in men, an Odds ratio >1 for dichotomous risk factors indicates a higher risk in the presence of the respective risk factor, while an Odds ratio >1 for continuous variables indicates a higher risk at higher values of the potential risk factor.

**SUPPLEMENTARY TABLE S4.** Multivariate statistical analyses of risk factors for functional outcomes at 90 days.

|  | **mRS^a^** | |  | **mRS 6 (death)^b^** | |
| --- | --- | --- | --- | --- | --- |
|  | **Common odds ratio (95% CI)** | ***P* value** |  | **Odds ratio (95% CI)** | ***P* value** |
| **Male/female** | 0.59 (0.36-1.00) | 0.052 |  | 0.41 (0.19-0.90) | **0.026** |
| **Age (years)** | 1.05 (1.03-1.08) | **<0.001** |  | 1.04 (1.00-1.09) | **0.041** |
| **TMT (mm)** | 0.99 (0.76-1.29) | 0.919 |  | 0.98 (0.64-1.49) | 0.914 |
| **NIHSS at admission** | 1.14 (1.09-1.19) | **<0.001** |  | 1.09 (1.03-1.15) | **0.002** |
| **ICH volume (ml)** | 1.01 (1.00-1.01) | 0.209 |  | 1.01 (1.00-1.02) | **0.034** |
| **Insular hemorrhage** | 1.24 (0.68-2.25) | 0.487 |  | 1.45 (0.65-3.20) | 0.364 |
| **Intraventricular hemorrhage (IVH)** | 3.63 (2.12-6.24) | **<0.001** |  | 1.99 (0.91-4.32) | 0.081 |
| **Accompanied SAH** | 1.46 (0.82-2.60) | 0.195 |  | 1.58 (0.73-3.46) | 0.249 |
| **Pneumonia** | 6.83 (3.82-12.22) | **<0.001** |  | 7.62 (3.62-16.07) | **<0.001** |
| **Atrial fibrillation** | 1.64 (0.86-3.10) | 0.127 |  | 1.81 (0.77-4.27) | 0.174 |
| **Anticoagulation** | 1.22 (0.61-2.44) | 0.576 |  | 1.42 (0.56-3.62) | 0.460 |
| **Hypertension** | 1.37 (0.38-4.95) | 0.635 |  | - | - |

ICH: intracranial hemorrhage; IVH: intraventricular hemorrhage; mRS: modified ranking scale; NIHSS: National Institute of Health Stroke Scale; SAH: subarachnoidal hemorrhage; TMT: temporalis muscle thickness.

^a^Odds ratios and *P* values from multivariate ordinal regression analyses using the variables resulted statistically significant in the univariate comparison as predictors. An Odds ratio >1 for sex as a risk factor indicates a higher risk for the outcome/death in men, an Odds ratio >1 for dichotomous risk factors indicates a higher risk in the presence of the respective risk factor, while an Odds ratio >1 for continuous variables indicates a higher risk at higher values of the potential risk factor.

^b^Odds ratios and *P* values from multivariate binary logistic regression analyses using the variables resulted statistically significant in the univariate comparison as predictors. An Odds ratio >1 for sex as a risk factor indicates a higher risk for the outcome/death in men, an Odds ratio >1 for dichotomous risk factors indicates a higher risk in the presence of the respective risk factor, while an Odds ratio >1 for continuous variables indicates a higher risk at higher values of the potential risk factor.

**SUPPLEMENTARY TABLE S5.** Univariate and multivariate statistical analyses of candidate risk factors for death within 7 days.

|  | **Univariate logistic regression^a^** | |  | **Multivariate logistic regression^b^** | |
| --- | --- | --- | --- | --- | --- |
|  | **Odds ratio (95% CI)** | ***P* value** |  | **Odds ratio (95% CI)** | ***P* value** |
| **Male/female** | 0.74 (0.40-1.37) | 0.339 |  | - | - |
| **Age (years)** | 1.03 (1.00-1.06) | **0.042** |  | 1.06 (1.02-1.11) | **0.004** |
| **BMI** | 0.98 (0.91-1.05) | 0.514 |  | - | - |
| **TMT (mm)** | 0.83 (0.62-1.11) | 0.201 |  | - | . |
| **Sarcopenia** | 1.31 (0.67-2.56) | 0.437 |  | - | - |
| **NIHSS at admission** | 1.19 (1.14-1.25) | **<0.001** |  | 1.15 (1.09-1.22) | **<0.001** |
| **ICH volume (ml)** | 1.03 (1.02-1.03) | **<0.001** |  | 1.01 (1.00-1.02) | **0.009** |
| **Insular hemorrhage** | 4.65 (2.44-8.87) | **<0.001** |  | 1.82 (0.79-4.21) | 0.162 |
| **Intraventricular hemorrhage (IVH)** | 9.25 (4.00-21.41) | **<0.001** |  | 2.13 (0.77-5.93) | 0.147 |
| **Accompanied SAH** | 1.37 (0.68-2.76) | 0.378 |  | - | - |
| **Surgical therapy** | 1.31 (0.48-2.38) | 0.261 |  | - | - |
| **Modified ICH score** | 2.33 (1.85-2.94) | **<0.001** |  | - | - |
| **Pneumonia** | 9.53 (3.84-18.98) | **<0.001** |  | 1.98 (0.75-5.25) | 0.167 |
| **Atrial fibrillation** | 1.00 (0.51-1.96) | 1.000 |  | - | - |
| **Anticoagulation** | 1.77 (0.91-3.45) | 0.092 |  | - | - |
| **Hypertension** | - | - |  | - | - |
| **Diabetes mellitus** | 1.14 (0.59-2.22) | 0.695 |  | - | - |

BMI: body mass index; ICH: intracranial hemorrhage; IVH: intraventricular hemorrhage; NIHSS: National Institute of Health Stroke Scale; SAH: subarachnoidal hemorrhage; TMT: temporalis muscle thickness.

^a^Odds ratios and *P* values from univariate binary logistic regression analyses. An Odds ratio >1 for sex as a risk factor indicates a higher risk for death in men, an Odds ratio >1 for dichotomous risk factors indicates a higher risk in the presence of the respective risk factor, while an Odds ratio >1 for continuous variables indicates a higher risk at higher values of the potential risk factor.

^b^Odds ratios and *P* values from multivariate binary logistic regression analyses using the variables resulted statistically significant in the univariate comparison as predictors. An Odds ratio >1 for sex as a risk factor indicates a higher risk for death in men, an Odds ratio >1 for dichotomous risk factors indicates a higher risk in the presence of the respective risk factor, while an Odds ratio >1 for continuous variables indicates a higher risk at higher values of the potential risk factor. To avoid structural collinearity, modified ICH score were not included into regression model, since score items were already included as separate covariates.

**SUPPLEMENTARY TABLE S6.** Univariate and multivariate Cox regression analyses of candidate risk factors for survival.

|  | **Univariate Cox regression^a^** | |  | **Multivariate Cox regression^b^** | |
| --- | --- | --- | --- | --- | --- |
|  | **Hazard ratio (95% CI)** | ***P* value** |  | **Hazard ratio (95% CI)** | ***P* value** |
| **Male/female** | 0.60 (0.41-0.88) | **0.009** |  | 0.64 (0.40-1.02) | 0.061 |
| **Age (years)** | 1.04 (1.02-1.05) | **<0.001** |  | 1.04 (1.01-1.06) | **0.002** |
| **BMI** | 0.96 (0.92-1.01) | 0.092 |  | - | - |
| **TMT (mm)** | 0.78 (0.65-0.94) | **0.006** |  | 0.89 (0.69-1.14) | 0.365 |
| **Sarcopenia** | 1.38 (0.92-2.07) | 0.124 |  | - | - |
| **NIHSS at admission** | 1.12 (1.10-1.15) | **<0.001** |  | 1.08 (1.05-1.14) | **<0.001** |
| **ICH volume (ml)** | 1.02 (1.01-1.02) | **<0.001** |  | 1.01 (1.00-1.01) | **<0.001** |
| **Insular hemorrhage** | 2.56 (1.74-3.77) | **<0.001** |  | 1.22 (0.79-1.87) | 0.366 |
| **Intraventricular hemorrhage (IVH)** | 3.94 (2.57-6.04) | **<0.001** |  | 1.62 (0.99-2.67) | 0.055 |
| **Accompanied SAH** | 1.70 (1.14-2.55) | **0.010** |  | 1.13 (0.74-1.73) | 0.584 |
| **Surgical therapy** | 1.24 (0.63-2.47) | 0.530 |  | - | - |
| **Modified ICH score** | 1.79 (1.61-1.99) | **<0.001** |  | - | - |
| **Pneumonia** | 8.44 (5.08-14.01) | **<0.001** |  | 3.22 (1.86-5.59) | **<0.001** |
| **Atrial fibrillation** | 1.95 (1.33-2.85) | **<0.001** |  | 0.88 (0.54-1.45) | 0.629 |
| **Anticoagulation** | 1.87 (1.26-2.77) | **0.002** |  | 1.58 (0.95-2.62) | 0.079 |
| **Hypertension** | - | - |  | - | - |
| **Diabetes mellitus** | 1.00 (0.66-1.51) | 0.981 |  | - | - |

BMI: body mass index; ICH: intracranial hemorrhage; IVH: intraventricular hemorrhage; NIHSS: National Institute of Health Stroke Scale; SAH: subarachnoidal hemorrhage; TMT: temporalis muscle thickness.

^a^Hazard ratios and *P* values from univariate Cox regression analyses. A Hazard ratio >1 for sex as a risk factor indicates a higher risk for death in men, aHazard ratio >1 for dichotomous risk factors indicates a higher risk in the presence of the respective risk factor, while a Hazard ratio >1 for continuous variables indicates a higher risk at higher values of the potential risk factor.

^b^Hazard ratios and *P* values from multivariate Cox regression analyses using the variables resulted statistically significant in the univariate comparison as predictors. A Hazard ratio >1 for sex as a risk factor indicates a higher risk for death in men, a Hazard ratio >1 for dichotomous risk factors indicates a higher risk in the presence of the respective risk factor, while a Hazard ratio >1 for continuous variables indicates a higher risk at higher values of the potential risk factor. To avoid structural collinearity, modified ICH score was not included into regression model, since score items were already included as separate covariates.

**Supplementary Figure S1**


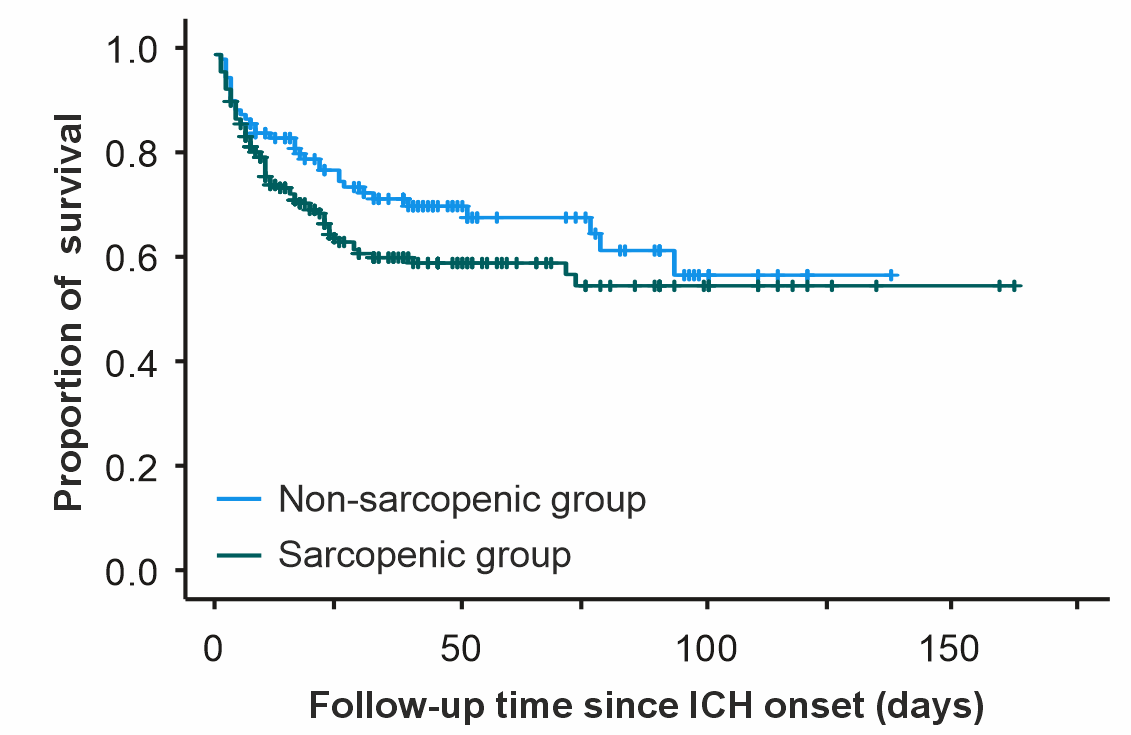


**SUPPLEMENTARY FIGURE S1. Kaplan-Meier curve showing the association between sarcopenia and survival after intracerebral hemorrhage (ICH).**

The Kaplan-Meier curve shows the survival of patients after intracerebral hemorrhage (ICH) grouped into non-sarcopenic and sarcopenic patients according to the temporalis muscle thickness (TMT) using published thresholds.
